# *Phylloporia lonicerae* (Hymenochaetales, Basidiomycota), a new species on *Lonicera japonica* from Japan and an identification key to worldwide species of *Phylloporia*

**DOI:** 10.3897/mycokeys.30.23235

**Published:** 2018-02-14

**Authors:** Wen-Min Qin, Xue-Wei Wang, Takuo Sawahata, Li-Wei Zhou

**Affiliations:** 1 Institute of Applied Ecology, Chinese Academy of Sciences, Shenyang 110016, China; 2 University of Chinese Academy of Sciences, Beijing 100049, China; 3 Faculty of Agriculture, Kindai University, 3327-204 Nakamachi, Nara 631-8505, Japan

**Keywords:** Hymenochaetaceae, key, *Lonicera japonica*, polypore, taxonomy

## Abstract

*Phylloporia*, in the Hymenochaetaceae, is a polypore genus with a worldwide distribution. The new taxon *Phylloporia lonicerae* is introduced, which is the first *Phylloporia* species to originate from Japan. This species grows exclusively on living *Lonicera japonica* and is distinguished by annual, sessile basidiocarps that occur in clusters, pileal surface of narrow, concentrically sulcate zones, 6–8 pores per mm, duplex context separated by a black zone, dimitic hyphal system and broadly ellipsoid basidiospores, 3.2–4 × 2.3–3.1 µm. Phylogenetically, *P. lonicerae* is nested within the *Phylloporia* clade as a distinct terminal lineage with full statistical supports and sister to the clade of *P. minutispora*, P. cf. pulla and *P. terrestris* with weak supports. Besides *Phylloporia bibulosa*, *P. chrysites* and *P. spathulata*, *P. lonicerae* is the fourth species of *Phylloporia* recorded from Japan. An identification key to all accepted 48 species of *Phylloporia* is provided.

## Introduction

*Phylloporia* Murrill, in the Hymenochaetaceae Donk, was introduced for an unusual polypore species, *P. parasitica* Murrill growing on the underside of living leaves in Columbia (Murrill 1904). For nearly 70 years, *Phylloporia* was forgotten until Ryvarden (1972) transferred five taxa into the genus. Renewed interest in *Phylloporia* was stimulated by Wagner and Ryvarden’s (2002) phylogenetic and morphological study in which they accepted 12 species. Since then, a number of new species have been described from Africa (Ipulet and Ryvarden 2005, Decock et al. 2015, Yombiyeni et al. 2015, Yombiyeni and Decock 2017), the Americas (Valenzuela et al. 2011, Decock et al. 2013, Ferreira-Lopes et al. 2016) and Asia, especially China (Gafforov et al. 2014, Cui et al. 2010, Zhou and Dai 2012, Zhou 2013, 2015a, 2015b, 2016, Liu et al. 2015, Chen et al. 2017, Ren and Wu 2017).

*Phylloporia* began as a monophyletic genus based on phylogenic studies of the large subunit of the nuclear ribosomal gene (nLSU) (Wagner and Ryvarden 2002) but is now paraphyletic with the inclusion of Coltricia cf. stuckertiana (Speg.) Rajchenb. & J.E. Wright in the *Phylloporia* clade (Valenzuela et al. 2011, Decock et al. 2013). The genus is morphologically quite diverse and includes species with annual or perennial basidiocarps with resupinate, sessile or stipitate habits, homogenous or duplex context, monomitic or dimitic hyphal system and cylindrical to subglobose basidiospores (Wagner and Ryvarden 2002, Cui et al. 2010, Zhou 2015a). Substrate preferences of *Phylloporia* species are equally diverse. Some species are saprobes that colonise woody debris (Ipulet and Ryvarden 2005, Zhou 2015b, Ferreira-Lopes et al. 2016) and others are parasites usually of specific plant hosts (Zhou 2015a, Ren and Wu 2017, Yombiyeni and Decock 2017).

There are three species of *Phylloporia* reported from Japan – *P. bibulosa* (Lloyd) Ryvarden, *P. chrysites* (Berk.) Ryvarden and *P. spathulata* (Hook.) Ryvarden (Núñez and Ryvarden 2000). In this paper, a new species, *Phylloporia lonicerae*, is described from Nara, Japan, growing on living vines of *Lonicera japonica*. Morphological and molecular data support the recognition of this new species. In addition, an updated key to the known species of *Phylloporia* is presented.

## Materials and methods

### Morphological examination

The studied specimens were deposited at the herbarium of the Institute of Applied Ecology, Chinese Academy of Sciences (IFP) in China. The macroscopic characters were observed from dried specimens with the aid of a stereomicroscope. Specimen sections were mounted in Cotton Blue (CB), Melzer’s reagent (IKI) and 5 % potassium hydroxide (KOH) for observation using a Nikon Eclipse 80i microscope at magnification up to 1000×. Special colour terms follow Petersen (1996). All measurements were taken from sections mounted in CB. When presenting the size variation of basidiospores, 5% of measurements from each end of the range were put in parentheses. Line drawings of microscopic characters were made with the aid of a drawing tube. The abbreviations used in the description are as follows: L = mean basidiospore length (arithmetic average of all measured basidiospores), W = mean basidiospore width (arithmetic average of all measured basidiospores), Q = variation in the L/W ratios between the specimens studied and n = number of basidiospores measured from a given number of specimens.

### Molecular sequencing

**Figure 1.**
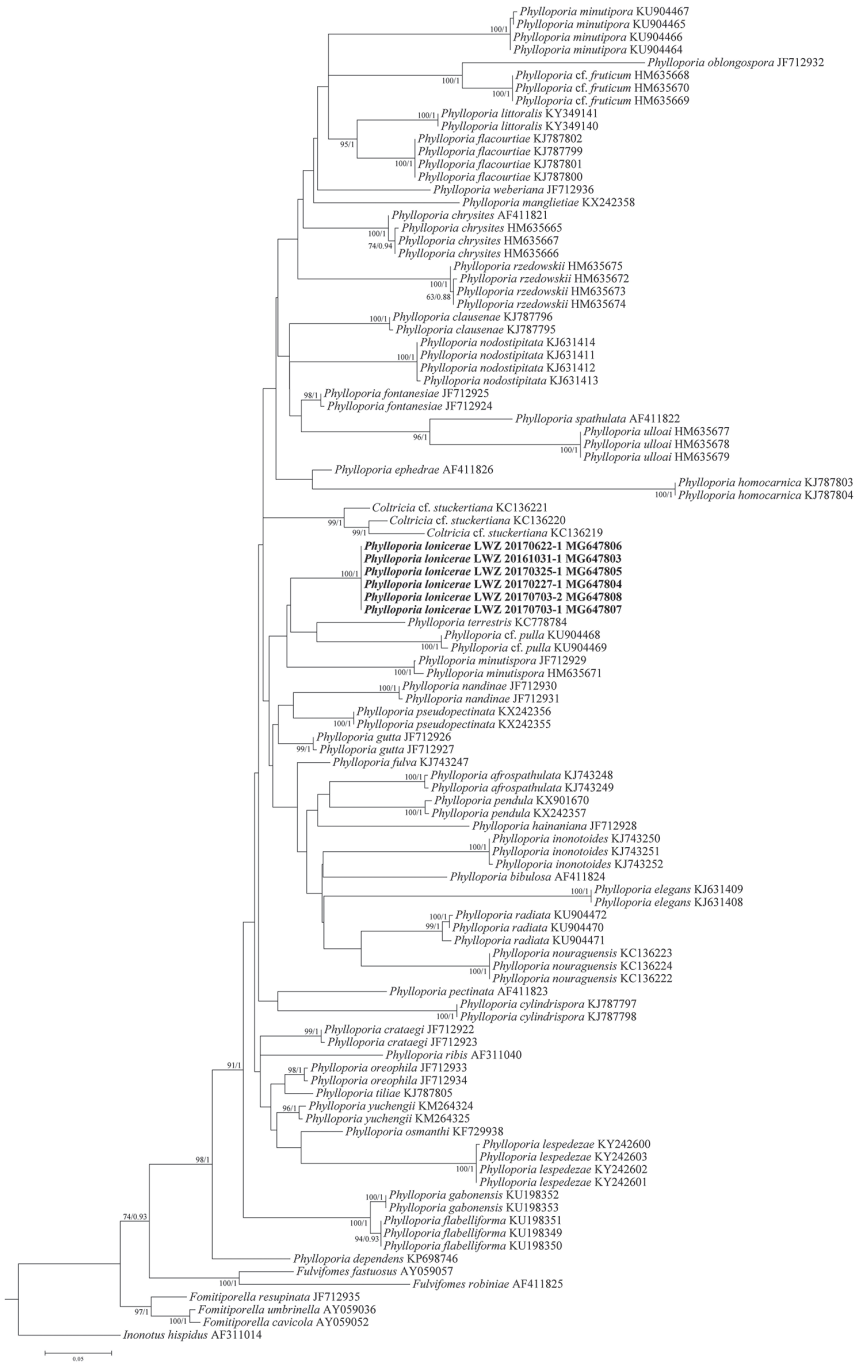
Phylogenetic position of *Phylloporia lonicerae* inferred from the nLSU dataset. The topology is inferred by maximum likelihood algorithm, while bootstrap values above 50 % and Bayesian posterior probabilities above 0.8 are given at the nodes. Newly sequenced specimens are in boldface.

The PCR products were directly amplified from the extracts of the basidiocarps with the Phire® Plant Direct PCR Kit (Finnzymes Oy, Finland) according to the manufacturer’s protocol. The PCR protocol was as follows: initial denaturation at 98 °C for 5 min, followed by 39 cycles of denaturation at 98 °C for 5 s, annealing at 48 °C for 5 s and extension at 72 °C for 5 s and a final extension of 72 °C for 10 min. The primers LR0R and LR7 (Vilgalys and Hester 1990) were used for PCR amplification and subsequent sequencing at the Beijing Genomics Institute, China. The newly generated sequences were submitted to GenBank (http://www.ncbi.nlm.nih.gov/genbank; Fig. 1).

### Phylogenetic analysis

To explore the phylogenetic relationship of *P. lonicerae*, six nLSU sequences were incorporated into previous nLSU datasets of *Phylloporia* (Zhou 2016, Chen et al. 2017, Ren and Wu 2017, Yombiyeni and Decock 2017). Several species of *Fomitiporella* Murrill and *Fulvifomes* Murrill were included in the dataset and *Inonotus hispidus* (Bull.) P. Karst. was selected as the outgroup taxon.

The nLSU dataset was aligned with MAFFT 7.110 (Katoh and Standley 2013) with the g-ini-i option (Katoh et al. 2005). The best-fit evolutionary model for the resulting alignment that was deposited in TreeBASE (http://www.treebase.org; accession number S21971), was estimated as GTR + I + G using jModelTest 2.1.4 (Darriba et al. 2012). Following this model, maximum likelihood (ML) and Bayesian Inference (BI) algorithms were used to infer the phylogeny of the alignment. The ML analysis was conducted using raxmlGUI 1.2 (Silvestro and Michalak 2012, Stamatakis 2006) under the auto FC option for bootstrap (BS) replicates (Pattengale et al. 2010). MrBayes 3.2 (Ronquist et al. 2012) was carried out for BI analysis, which employed two independent runs, each including four chains of 10 million generations and starting from random trees. Trees were sampled every 1000th generation. Of the sampled trees, the first 25 % was deleted and the remaining trees were used to construct a 50 % majority consensus tree and calculate Bayesian posterior probabilities (BPPs). Chain convergence was determined using Tracer 1.5 (http://tree.bio.ed.ac.uk/software/tracer/).

## Results

Six nLSU sequences of *P. lonicerae* were generated and included in a dataset of 105 sequences and 942 characters. ML analysis was ended after 250 BS replicates. BI analysis converged all chains as indicated by the effective sample sizes of all parameters above 2000 and the potential scale reduction factors close to 1.000. As the ML and BI analyses generated congruent topologies in main lineages, the ML tree is presented in Figure 1. Values of BS above 50 % and BPPs above 0.8 are given at the nodes. The phylogenic tree (Fig. 1) shows that the strongly supported *Phylloporia* clade (98 % in ML, 1 in BI) consists of 44 terminal lineages and the six *P. lonicerae* samples formed a new lineage with full statistical supports (100 % in ML, 1 in BI). The *Phylloporia lonicerae* lineage is sister to the clade that includes *P. minutispora* Ipulet & Ryvarden, P. cf. pulla (Mont. & Berk.) Decock & Yombiy and *P. terrestris* L.W. Zhou with weak supports.

## Taxonomy

### Phylloporia lonicerae

Taxon classificationFungiHymenochaetalesHymenochaetaceae

W.M. Qin, Xue W. Wang, T. Sawahata & L.W. Zhou sp. nov.

MB823715

Figs 2 , 3

#### Holotype.

JAPAN. Nara, Research Forest of Faculty of Agriculture, Kindai University, 3 Jul 2017, on living vine of *Lonicera japonica*, LWZ 20170703-2 (IFP 019172).

#### Etymology.

*Lonicerae* (Lat.): referring to *Lonicera*, the host tree genus.

Basidiocarps annual, sessile, imbricate, rarely solitary, without odour or taste, woody. Pilei semi-circular, flabelliform or fused together, applanate, single pileus projecting up to 1.5 cm long, 3 cm wide and 0.5 cm thick at base. Pileal surface greyish-brown to yellowish-brown, velutinate, concentrically sulcate with narrow zones; margin pale yellow or concolorous, sharp. Pore surface honey-yellow, slightly glancing; sterile margin distinct, curry-yellow, up to 0.5 mm wide; pores circular to angular, 6–8 per mm; dissepiments thin, entire. Context up to 2 mm thick, duplex, with a black zone, lower context olivaceous buff, hard corky, up to 1 mm thick, upper tomentum cinnamon-buff, soft, up to 1 mm thick. Tubes honey-yellow, corky, up to 3 mm long.

**Figure 2.**
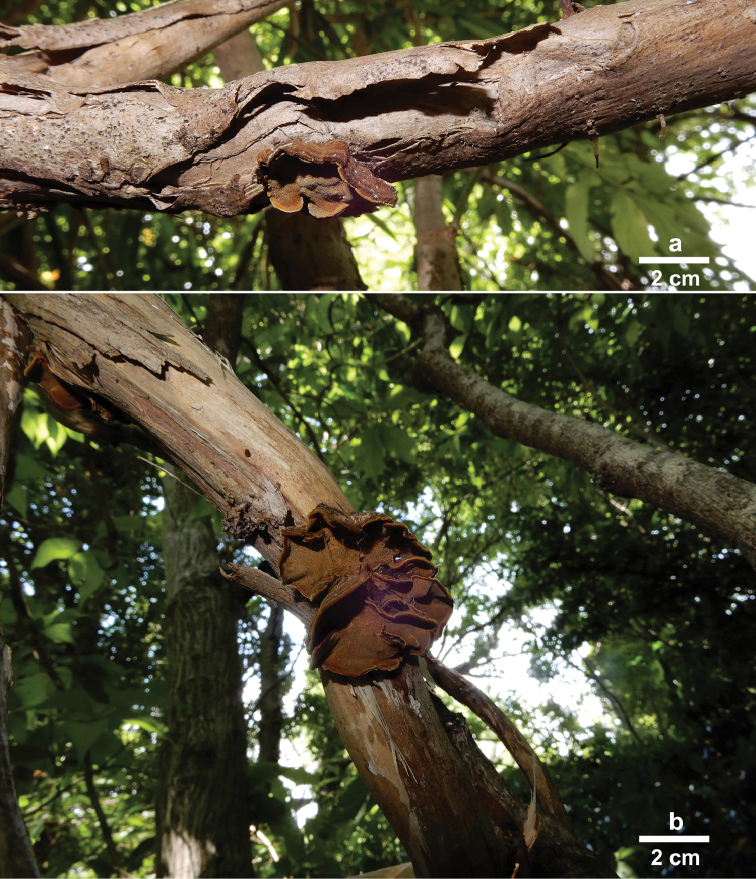
Basidiocarps of *Phylloporia lonicerae* in situ. **a** LWZ 20170703-2 (holotype) **b** LWZ 20170622-1 (paratype). Scale bars: 2 cm.

**Figure 3.**
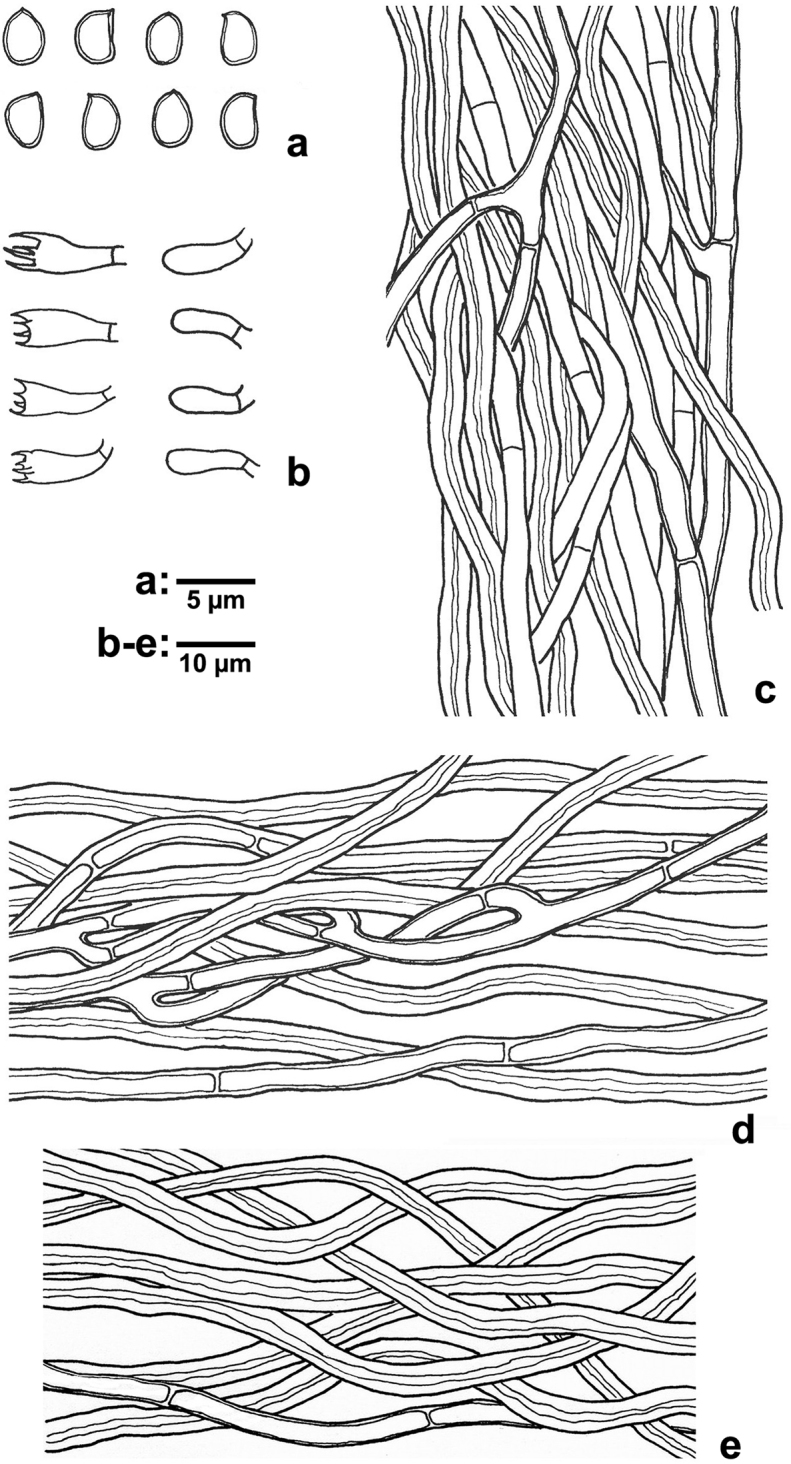
Microscopic structures of *Phylloporia lonicerae* (drawn from the holotype, LWZ 20170703-2). **a** Basidiospores **b** Basidia and basidioles **c** Hyphae from trama **d** Hyphae from lower context **e** hyphae from upper tomentum. Scale bars: **a** = 5 µm, **b–e** = 10 µm.

Hyphal system dimitic; generative hyphae simple septate; tissue darkening but otherwise unchanged in KOH. Context: in the lower context, generative hyphae hyaline to pale yellowish, slightly thick- to thick-walled with a wide lumen, frequently branched and septate, 2–4 μm in diam; skeletal hyphae golden yellow, thick-walled with a narrow lumen, unbranched, aseptate, interwoven, 2–4.5 μm in diam; in the upper tomentum, generative hyphae infrequent, pale yellowish, slightly thick- to thick-walled with a wide lumen, rarely branched, frequently septate, 2–4 μm in diam; skeletal hyphae golden yellow, thick-walled with a narrow to wide lumen, unbranched, aseptate, loosely interwoven, 2.5–5 μm in diam; in the black zone, hyphae dark brown, thick-walled with a narrow lumen, strongly agglutinated, interwoven. Tubes: generative hyphae hyaline to pale yellowish, thin- to slightly thick-walled, occasionally branched, frequently septate, 1.8–4 μm in diam; skeletal hyphae golden yellow, thick-walled with a narrow lumen, unbranched, aseptate, interwoven, 2–4 μm in diam. Setae absent. Cystidia and cystidioles absent. Basidia clavate, with four sterigmata up to 3 μm long and a simple septum at the base, 7–11 × 4–6 μm; basidioles in shape similar to basidia, but slightly smaller. Basidiospores broadly ellipsoid, pale yellowish, thick-walled, smooth, indextrinoid, inamyloid, acyanophilous, (3–)3.2–4 × (2.1–)2.3–3.1(–3.3) μm, L = 3.61 μm, W = 2.77 μm, Q = 1.28–1.33 (n = 90/3).

#### Additional specimens (paratypes) studied.

(All on living vine of *Lonicera japonica*)—JAPAN. Nara, Research Forest of Faculty of Agriculture, Kindai University, 31 Oct 2016, LWZ 20161031-1 (IFP 019173); 27 Feb 2017, LWZ 20170227-1 (IFP 019174); 25 Mar 2017, LWZ 20170325-1 (IFP 019175); 22 Jun 2017, LWZ 20170622-1 (IFP 019176); 3 Jul 2017, LWZ 20170703-1 (IFP 019177).

## Discussion

*Phylloporia lonicerae* is morphologically distinct from other species in *Phylloporia* by its annual, sessile basidiocarps that occur in clusters, pileal surface of narrow, concentrically sulcate zones, 6–8 pores per mm, duplex context separated by a black zone, dimitic hyphal system and broadly ellipsoid basidiospores, 3.2–4 × 2.3–3.1 µm. In the field, it is readily identified by fruiting on living vines, >1.5 cm diameter, of *Lonicera japonica*. *Phylloporia lonicerae* is most similar to *P. pseudopectinata* Yuan Y. Chen & B.K. Cui and *P. minutipora* L.W. Zhou by sharing annual, sessile basidiocarps in clusters and a dimitic hyphal system, but easily distinguished from *P. pseudopectinata* by larger pores (8–9 per mm) and subglobose basidiospores (Chen et al. 2017) and from *P. minutipora* by larger pores and basidiospores and the specific host (Zhou 2016). An updated key, based on Zhou (2016), to all accepted 48 species of *Phylloporia* is provided below.

*Lonicera japonica* is a well-known and important medicinal plant (Li 1578). Therefore, the potential medicinal applications of fungi growing on this plant are intriguing. Li et al. (2010) studied the medicinal metabolites from basidiocarps of *Phylloporia ribis* (Schumach.) Ryvarden that were collected on *Lonicera japonica* in China. Recent phylogenetic evidence, however, indicates that Chinese specimens of *P. ribis* collected on hosts other than *Ribes* are distinct from a *P. ribis* specimen collected on *Ribes* in Germany (Zhou and Dai 2012). As *P. ribis* was originally described from Denmark (Larsen and Cobb-Poulle 1990), *P. ribis* specimens used by Li et al. (2010) in their study are likely *P. lonicerae* or another undescribed species.

Some species of *Phylloporia* are parasitic and appear to be restricted by host and geographic distribution of its host. For example, *Phylloporia crataegi* L.W. Zhou & Y.C. Dai, which occurs exclusively on living *Crataegus* and *P. fontanesiae* L.W. Zhou & Y.C. Dai, which colonises living *Fontanesia*, are widely distributed in China (Zhou and Dai 2012, unpublished data). Similarly, in central African rainforests, *P. flabelliformis* Decock & Yombiy is found on living trunks of *Dichostemma* and *Anthostema* whereas *P. gabonensis* Decock & Yombiy occurs only on *Dichostemma* (Decock et al. 2015). In contrast, *Lonicera japonica* has a worldwide distribution and so far is host to a single species of *Phylloporia*. It will be interesting to determine if *P. lonicerae* is found elsewhere on *Lonicera japonica* or if different species of *Phylloporia* are found on living *Lonicera japonica* in other geographic regions.

Since 2010, 21 new species of *Phylloporia* have been described from China (Cui et al. 2010, Zhou and Dai 2012, Zhou 2013, 2015a, 2015b, 2016, Liu et al. 2015, Chen et al. 2017, Ren and Wu 2017). Yet in Japan, only four *Phylloporia* species, including *P. lonicerae*, are known. It is hoped that this paper will draw attention to this genus in Japan and lead to the discovery of additional species.

### Key to worldwide species of *Phylloporia*

**Table:** 

1	Basidiocarps resupinate	***P. parasitica***
–	Basidiocarps sessile or stipitate	**2**
2	Basidiocarps stipitate and terrestrial (woody debris)	**3**
–	Basidiocarps sessile and on aerial wood	**9**
3	Context homogeneous	***P. minutispora***
–	Context duplex	**4**
4	Basidiospores > 4 µm long, > 3 µm wide	***P. verae-crucis* (Berk. ex Sacc.) Ryvarden**
–	Basidiospores < 4 µm long, < 3 µm wide	**5**
5	Cystidia present	**6**
–	Cystidia absent	**7**
6	Hyphae in tomentum short and anticlinal	***P. elegans* Ferreira-Lopes, Robledo & Drechsler-Santos**
–	Hyphae in tomentum loosely interwoven	***P. nodostipitata* Ferreira-Lopes & Drechsler-Santos**
7	Pores < 10 per mm	***P. spathulata***
–	Pores > 10 per mm	**8**
8	Basidiospores < 3.3 µm long, < 2.3 µm wide	***P. terrestris***
–	Basidiospores > 3.3 µm long, > 2.3 µm wide	***P. afrospathulata* Yombiy & Decock**
9	Hyphal system dimitic	**10**
–	Hyphal system monomitic	**18**
10	Basidiocarps perennial	**11**
–	Basidiocarps annual	**12**
11	Pores 6–8 per mm	***P. manglietiae* Yuan Y. Chen & B.K. Cui**
–	Pores 8–11 per mm	***P. pectinata* (Klotzsch) Ryvarden**
12	Basidiocarps solitary	***P. nouraguensis* Decock & G. Castillo**
–	Basidiocarps in cluster	**13**
13	Pileal surface lighter (greyish-orange to pale cinnamon)	***P. fulva* Yombiy & Decock**
–	Pileal surface darker (yellowish-brown to dark brown)	**14**
14	Pileus attached by a small vertex and pendant	**15**
–	Pileus widely attached to the substratum	**16**
15	Pores 7–9 per mm; basidiospores > 3.5 µm long	***P. pendula* Yuan Y. Chen & B.K. Cui**
–	Pores 11–12 per mm; basidiospores < 3.5 µm long	***P. pulla***
16	Pores 12–15 per mm; basidiospores < 3 µm long, < 2.5 µm wide	***P. minutipora***
–	Pores 6–9 per mm; basidiospores > 3 µm long, > 2.5 µm wide	**17**
17	Pores 6–9 per mm; basidiospores broadly ellipsoid (Q = 1.28–1.33)	***P. lonicerae***
–	Pores 8–9 per mm; basidiospores subglobose (Q = 1.21–1.23)	***P. pseudopectinata* Yuan Y. Chen & B.K. Cui**
18	Pores 2–4 per mm	**19**
–	Pores 4–12 per mm	**22**
19	Basidiospores broadly ellipsoid to subglobose	***P. fruticum* (Berk. & M.A. Curtis) Ryvarden**
–	Basidiospores oblong-ellipsoid, subcylindrical to cylindrical	**20**
20	Context duplex	***P. rzedowskii* R. Valenz. & Decock**
–	Context homogeneous	**21**
21	Context < 1 mm thick; on living branch	***P. oblongospora* Y.C. Dai & H.S. Yuan**
–	Context 2–4 mm thick; on living trunk	***P. inonotoides* Yombiy & Decock**
22	Basidiocarps annual to perennial, dense and hard consistency	**23**
–	Basidiocarps annual, soft corky at least at tomentum layer	**29**
23	Pores 10–12 per mm; on living *Tilia*	***P. tiliae* L.W. Zhou**
–	Pores 6–9 per mm; on other angiosperms	**24**
24	Pileal surface zonate and sulcate	**25**
–	Pileal surface azonate	***P. yuchengii* Gafforov, Tomšovský, Langer & L.W. Zhou**
25	Pores 6–7 per mm	**26**
–	Pores 7–9 per mm	**27**
26	Basidiospores ellipsoid; mostly on *Ribes*	***P. ribis***
–	Basidiospores subglobose; mostly on *Ephedra*, *Cotoneaster* or *Jasminum*	***P. ephedrae* (Woron.) Parmasto**
27	Basidiospores > 2.7 µm wide	***P. dependens* Y.C. Dai**
–	Basidiospores < 2.7 µm wide	**28**
28	Basidiospores ellipsoid to oblong-ellipsoid with a guttule; on *Abelia*	***P. gutta* L.W. Zhou & Y.C. Dai**
–	Basidiospores broadly ellipsoid without a guttule; on living *Crataegus*	***P. crataegi***
29	Basidiospores broadly ellipsoid to subglobose	**30**
–	Basidiospores ellipsoid, oblong-ellipsoid to cylindrical	**40**
30	Pores 5–6 per mm	**31**
–	Pores 6–11 per mm	**35**
31	Context duplex, separated by a black zone	**32**
–	Context not separated by a black zone	**33**
32	Pileal surface azonate, lower context 1–4 µm thick	***P. ampelina* (Bondartsev & Singer) Bondartseva**
–	Pileal surface zonate and sulcate, lower context 1 µm thick	***P. weberiana* (Bres. & Henn. ex Sacc.) Ryvarden**
33	Basidiocarps solitary covered by a thick tomentum layer, pileal surface not radially faintly wrinkled	***P. littoralis* Decock & Yombiy**
–	Basidiocarps in cluster without a distinct tomentum layer, pileal surface radially faintly wrinkled	**34**
34	Pileus < 1.5 mm thick, margin regular	***P. flabelliformis***
–	Pileus > 1.5 mm thick, margin irregular	***P. gabonensis***
35	Basidiocarps > 8 cm wide, > 15 mm thick; contextual hyphae > 5 µm in diam	***P. ulloai* R. Valenz., Raymundo, Cifuentes & Decock**
–	Basidiocarps < 8 cm wide, < 15 mm thick; contextual hyphae < 5 µm in diam	**36**
36	Contextual hyphae regularly arranged	**37**
–	Contextual hyphae interwoven	**38**
37	Pileus distinctly sulcate, not radially striate, margin obtuse, basal context separated by two black zones; hyphae in tomentum > 4 μm in diam; on living angiosperm trunk	***P. clausenae* L.W. Zhou**
–	Pileus faintly sulcate, radially striate, margin sharp, context duplex thoroughly; hyphae in tomentum < 4 μm in diam; on living liana	***P. radiata* L.W. Zhou**
38	Contextual hyphae slightly thick-walled with a wide lumen, frequently septate, large rhomboid crystals absent	**39**
–	Contextual hyphae thick-walled with a narrow lumen, occasionally septate, large rhomboid crystals present in trama and context	***P. chrysites***
39	Pores 10–12 per mm; basidiospores < 3 µm long; on living *Fontanesia*	***P. fontanesiae***
–	Pores 7–9 per mm; basidiospores > 3 µm long; on other angiosperms	***P. oreophila* L.W. Zhou & Y.C. Dai**
40	Basidiospores mostly > 3 µm wide	**41**
–	Basidiospores mostly < 3 µm wide	**42**
41	Pores 4–6 per mm	***P. hainaniana* Y.C. Dai & B.K. Cui**
–	Pores 8–10 per mm	***P. capucina* (Mont.) Ryvarden**
42	Basidiocarp solitary	**43**
–	Basidiocarp imbricate	**46**
43	Context homogeneous	***P. homocarnica* L.W. Zhou**
–	Context duplex	**44**
44	Context not separated by a black zone; on living *Flacourtia*	***P. flacourtiae* L.W. Zhou**
–	Context separated by a black zone; on other angiosperms	**45**
45	Pileal surface azonate, pores 6–8 per mm; basidiospores cylindrical	***P. cylindrispora* L.W. Zhou**
–	Pileal surface zonate and sulcate, pores 8–9 per mm; basidiospores ellipsoid	***P. lespedezae* G.J. Ren & F. Wu**
46	Basidiospores mostly < 2.5 µm wide	**47**
–	Basidiospores mostly > 2.5 µm wide	***P. bibulosa***
47	Pores 5–6 per mm, context duplex, not separated by a black zone; basidiospores > 3.5 µm long, contextual hyphae interwoven; on living *Nandina*	***P. nandinae* L.W. Zhou & Y.C. Dai**
–	Pores 7–9 per mm, context duplex, separated by a black zone; basidiospores < 3.5 µm long, contextual hyphae regularly arranged; on living *Osmanthus*	***P. osmanthi* L.W. Zhou**

## Supplementary Material

XML Treatment for Phylloporia lonicerae

## References

[B1] Chen YY Zhu L Xing JH Cui BK (2017) Three new species of *Phylloporia* (Hymenochaetales) with dimitic hyphal systems from tropical China. Mycologia. https://doi.org/10.1080/00275514.2017.1410692 10.1080/00275514.2017.1410692 29474112

[B2] Cui BK Yuan HS Dai YC (2010) Two new species of *Phylloporia* (Basidiomycota, Hymenochaetaceae) from China. Mycotaxon 113: 171–178. https://doi.org/10.5248/113.171

[B3] Darriba D Taboada GL Doallo R Posada D (2012) jModelTest 2: more models, new heuristics and parallel computing. Nature Methods 9: 772. https://doi.org/10.1038/nmeth.2109 10.1038/nmeth.2109 PMC4594756 22847109

[B4] Decock C Amalfi M Robledo G Castillo G (2013) *Phylloporia nouraguensis*, an undescribed species on Myrtaceae from French Guiana. Cryptogamie Mycologie 34: 15–27. https://doi.org/10.7872/crym.v34.iss1.2013.15

[B5] Decock C Yombiyeni P Memiaghe H (2015) Hymenochaetaceae from the Guineo-Congolian rainforest: *Phylloporia flabelliforma* sp. nov. and *Phylloporia gabonensis* sp. nov., two undescribed species from Gabon. Cryptogamie Mycologie 36: 449–467. https://doi.org/10.7872/crym/v36.iss4.2015.449

[B6] Ferreira-Lopes V Robledo GL Reck MA Neto AG (2016) *Phyllop*oria spath*ulata* sensu stricto and two new South American stipitate species of *Phylloporia* (Hymenochaetaceae). Phytotaxa 257: 133–148. https://doi.org/10.11646/phytotaxa.257.2.3

[B7] Gafforov Y Tomšovský M Langer E Zhou LW (2014) *Phylloporia yuchengii* sp. nov. (Hymenochaetales, Basidiomycota) from Western Tien Shan Mountains of Uzbekistan based on phylogeny and morphology. Cryptogamie Mycologie 35: 313–322. https://doi.org/10.7872/crym.v35.iss4.2014.313

[B8] Ipulet P Ryvarden L (2005) New and interesting polypores from Uganda. Synopsis Fungorum 20: 87–89.

[B9] Katoh K Kuma K Toh H Miyata T (2005) MAFFT version 5: improvement in accuracy of multiple sequence alignment. Nucleic Acids Research 33: 511–518. https://doi.org/10.1093/nar/gki198 15661851 10.1093/nar/gki198 PMC548345

[B10] Katoh K Standley DM (2013) MAFFT multiple sequence alignment software version 7: improvements in performance and usability. Molecular Biology and Evolution 30: 772–780. https://doi.org/10.1093/molbev/mst010 23329690 10.1093/molbev/mst010 PMC3603318

[B11] Larsen M Cobb-Poulle LA (1990) *Phellinus* (Hymenochaetaceae). A survey of the world taxa. Synopsis Fungorum 3: 1–206.

[B12] Li C Zhang YQ Li J Qiu LL (2010) Chemical constituents from fruiting bodies of *Phylloporia ribis* (*Lonicera japonica* Thunb.). Natural Product Research and Development 22: 422–424. [In Chinese]

[B13] Li SZ (1578) Compendium materia medica. Reprinted in 1957. Commercial Press, Beijing.

[B14] Liu JK Hyde KD Jones EBG Ariyawansa HA Bhat DJ Boonmee S Maharachchikumbura SSN McKenzie EHC Phookamsak R Phukhamsakda C Shenoy BD Abdel-Wahab MA Buyck B Chen J Chethana KWT Singtripop C Dai DQ Dai YC Daranagama DA Dissanayake AJ Doilom M D’souza MJ Fan XL Goonasekara ID Hirayama K Hongsanan S Jayasiri SC Jayawardena RS Karunarathna SC Li WJ Mapook A Norphanphoun C Pang KL Perera RH Peršoh D Pinruan U Senanayake IC Somrithipol S Suetrong S Tanaka K Thambugala KM Tian Q Tibpromma S Udayanga D Wijayawardene NN Wanasinghe D Wisitrassameewong K Zeng XY Abdel-Aziz FA Adamčík S Bahkali AH Boonyuen N Bulgakov T Callac P Chomnunti P Greiner K Hashimoto A Hofstetter V Kang JC Lewis D Li XH Liu XZ Liu ZY Matsumura M Mortimer PE Rambold G Randrianjohany E Sato G Sri-Indrasutdhi V Tian CM Verbeken A von Brackel W Wang Y Wen TC Xu JC Yan JY Zhao RL Camporesi E (2015) Fungal diversity notes 1–110: taxonomic and phylogenetic contributions to fungal species. Fungal Diversity 72: 1–197. https://doi.org/10.1007/s13225-015-0324-y

[B15] Murrill WA (1904) A new polyporoid genus from South America. Torreya 4: 141–142.

[B16] Núñez M Ryvarden L (2000) East Asian polypores Volume 1. Ganodermataceae and Hymenochaetaceae. Synopsis Fungorum 13: 1–168.

[B17] Pattengale ND Alipour M Bininda-Emonds ORP Moret BME Stamatakis A (2010) How many bootstrap replicates are necessary? Journal of Computational Biology 17: 337–354. https://doi.org/10.1089/cmb.2009.0179 10.1089/cmb.2009.0179 20377449

[B18] Petersen JH (1996) Farvekort. The Danish Mycological Society’s colour chart. Foreningen til Svampekundskabens Fremme, Greve.

[B19] Ren GJ Wu F (2017) *Phylloporia lespedezae* sp. nov. (Hymenochaetaceae, Basidiomycota) from China. Phytotaxa 299: 243–251. https://doi.org/10.11646/phytotaxa.299.2.8

[B20] Ronquist F Teslenko M van der Mark P Ayres D Darling A Höhna S Larget B Liu L Suchard MA Huelsenbeck JP (2012) MrBayes 3.2: Efficient Bayesian phylogenetic inference and model choice across a large model space. Systematic Biology 61: 539–542. https://doi.org/10.1093/sysbio/sys029 22357727 10.1093/sysbio/sys029 PMC3329765

[B21] Ryvarden L (1972) A critical checklist of the Polyporaceae in tropical East Africa. – Norwegian Journal of Botany 19: 229–238.

[B22] Silvestro D Michalak I (2012) raxmlGUI: a graphical front end for RAxML. Organisms Diversity & Evolution 12: 335–337. https://doi.org/10.1007/s13127-011-0056-0

[B23] Stamatakis A (2006) RAxML-VI-HPC: maximum likelihood-based phylogenetic analyses with thousands of taxa and mixed models. Bioinformatics 22: 2688–2690. https://doi.org/10.1093/bioinformatics/btl446 16928733 10.1093/bioinformatics/btl446

[B24] Valenzuela R Raymundo T Cifuentes J Castillo G Amalfi M Decock C (2011) Two undescribed species of *Phylloporia* from Mexico based on morphological and phylogenetic evidence. Mycological Progress 10: 341–349. https://doi.org/10.1007/s11557-010-0707-0

[B25] Vilgalys R Hester M (1990) Rapid genetic identification and mapping of enzymatically amplified ribosomal DNA from several *Cryptococcus* species. Journal of Bacteriology 172: 4238–4246. https://doi.org/10.1128/jb.172.8.4238-4246.1990 2376561 10.1128/jb.172.8.4238-4246.1990 PMC213247

[B26] Wagner T Ryvarden L (2002) Phylogeny and taxonomy of the genus *Phylloporia* (Hymenochaetales). Mycological Progress 1: 105–116. https://doi.org/10.1007/s11557-006-0009-8

[B27] Yombiyeni P Balezi A Amalfi M Decock C (2015) Hymenochaetaceae from the Guineo-Congolian rainforest: three new species of *Phylloporia* based on morphological, DNA sequences and ecological data. Mycologia 107: 996–1011. https://doi.org/10.3852/14-298 26240304 10.3852/14-298

[B28] Yombiyeni P Decock C (2017) Hymenochaetaceae (Hymenochaetales) from the Guineo-Congolian phytochorion: *Phylloporia littoralis* sp. nov. from coastal vegetation in Gabon, with an identification key to the local species. Plant Ecology and Evolution 150: 160–172. https://doi.org/10.5091/plecevo.2017.1289

[B29] Zhou LW (2013) https://doi.org/10.5248/124.361

[B30] Zhou LW (2015a) Four new species of *Phylloporia* (Hymenochaetales,Basidiomycota) from tropical China with a key to *Phylloporia* species worldwide. Mycologia 107: 1184–1192. https://doi.org/10.3852/14-254 26297774 10.3852/14-254

[B31] Zhou LW (2015b) *Phylloporia osmanthi* and *P. terrestris* spp. nov. (Hymenochaetales, Basidiomycota) from Guangxi, South China. Nova Hedwigia 100: 239–249. https://doi.org/10.1127/nova_hedwigia/2014/0220

[B32] Zhou LW (2016) *Phylloporia minutipora* and *P. radiata* spp. nov. (Hymenochaetales, Basidiomycota) from China and a key to worldwide species of *Phylloporia* Mycological Progress 15: 57. https://doi.org/10.1007/s11557-016-1200-1

[B33] Zhou LW Dai YC (2012) Phylogeny and taxonomy of *Phylloporia* (Hymenochaetales): new species and a worldwide key to the genus. Mycologia 104: 211–222. https://doi.org/10.3852/11-093 21933921 10.3852/11-093

